# Dynamics of the DNA Viral Community in Korean Coastal Waters

**DOI:** 10.1038/s41597-025-06062-w

**Published:** 2025-11-13

**Authors:** Yu Jin Kim, Kang Eun Kim, Hyun-Jung Kim, Joon Sang Park, Min-Jeong Kim, Seon Min Kim, Taehee Lee, Seung Won Jung

**Affiliations:** 1 Library of Marine Samples, Korea Institute of Ocean Science & Technology, Geoje, 53211 Republic of Korea; 2 Department of Ocean Science, University of Science and Technology, Daejeon, 34113 Republic of Korea; 3 Department of Oceanography and Marine Research Institute, Pusan National University, Busan, 46241 Republic of Korea; 4 Trophical & Subtrophical Research Center, Korea Institute of Ocean Science and Technology, Jeju, 63349 Republic of Korea

**Keywords:** Metagenomics, Marine biology

## Abstract

Recent advances in metaviromics have revealed vast viral diversity across aquatic environments, yet coastal marine viromes remain underexplored compared to their open-ocean counterparts. In this study, we analyzed 49 surface water samples from 16 coastal sites around Korea, generating 265 gigabases of metagenomic sequence data. Following quality control, 754 DNA viral contigs of ≥10 kb (medium quality or higher) were recovered, with bacteriophages comprising 95% and nucleocytoplasmic large DNA viruses (NCLDVs) 5% of the total. Among these, *Puniceispirillum* phage HMO-2011 and *Micromonas pusilla* virus 12 T exhibited the highest relative abundance within their respective groups. In addition, we provided the dataset of environmental parameters such as water temperature, salinity, etc., as well as viral taxonomic profiling of contig-level metadata. This dataset provides a resource for the investigation of coastal DNA viral communities and supports comparative studies across marine environments.

## Background & Summary

Viruses represent the most numerous biological entities on Earth, with their total population estimated to surpass 10^30^ globally^1^. They lyse hosts, providing various nutrients for the marine environment and contributing to biogeochemical cycles in marine ecosystems^2^. Recent studies estimate that bacteriophages lyse approximately 20%–40% of bacterial populations daily, highlighting their pivotal role in shaping microbial community structure and function^3,4^. Their prevalence is strongly associated with the high abundance of their bacterial hosts, including *Alphaproteobacteria* and *Cyanobacteria*^5,6^. Marine viruses are ubiquitous, from the surface to the bottom sediments of the ocean, making them the most abundant biological entities in aquatic ecosystems^7^. However, the diversity of viral communities in coastal waters and their relationships with environmental factors remain comparatively unknown. Coastal ecosystems are ecologically and economically vital, yet highly susceptible to anthropogenic and climate-induced stressors such as eutrophication, pollution, marine heatwaves, and harmful algal blooms (HABs), all of which can profoundly alter viral community structure and function^8^. Virome studies in these environments are therefore crucial for elucidating how viruses respond to and shape ecosystem dynamics under such pressures.

Viruses are broadly classified into DNA and RNA types, and their classification has advanced through integration of genomic and replication-based systems. The Baltimore classification, originally proposed in the 1970s, groups viruses into seven categories based on nucleic acid type (DNA or RNA), strandedness, sense, and replication strategy^9^. While still widely cited as a conceptual model, it has been refined by advances in genomic data. According to the most recent taxonomy from the International Committee on Taxonomy of Viruses^10^, viruses are now organized hierarchically based on evolutionary relationships inferred from genome sequences. In marine ecosystems, DNA viruses are especially dominant and play critical roles in host mortality, nutrient cycling, and microbial community structure^11^. The two major groups are bacteriophages and nucleocytoplasmic large DNA viruses (NCLDVs)^12,13^. Bacteriophages are primarily classified under the class *Caudoviricetes*, which includes families such as *Autographiviridae*, *Straboviridae*, *Herelleviridae*, and *Drexlerviridae*—all commonly detected in marine viromes^14–16^. NCLDVs infect a broad range of eukaryotic hosts, from unicellular protists to multicellular algae and metazoans^17,18^. Notably, NCLDVs predominantly infect autotrophic eukaryotes, such as haptophytes, chlorophytes, and dinoflagellates, playing a significant role in regulating primary production^5,19,20^. Major families within this group include *Mimiviridae* (formerly *Megaviridae*), *Phycodnaviridae*, *Pandoraviridae*, *Poxviridae*, *Iridoviridae*, *Marseilleviridae*, *Pithoviridae*, *Ascoviridae*, *Asfarviridae*, and *Mininucleoviridae*^21^. While numerous studies have underscored the ecological significance of both bacteriophages and NCLDVs, particularly in structuring microbial food webs, regulating host mortality, and influencing global biogeochemical cycles^22,23^, their diversity, functional capacities, and host interactions across various marine environments remain insufficiently characterized.

**Fig. 1:**
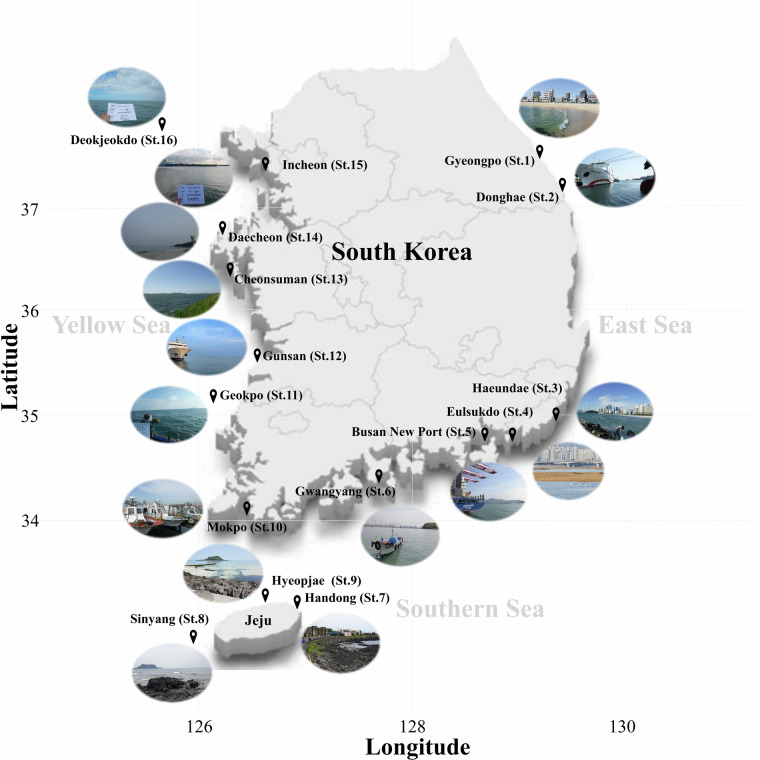
Map depicting the 16 sampling sites in the coastal waters of the Republic of Korea in 2021.

**Fig. 2:**
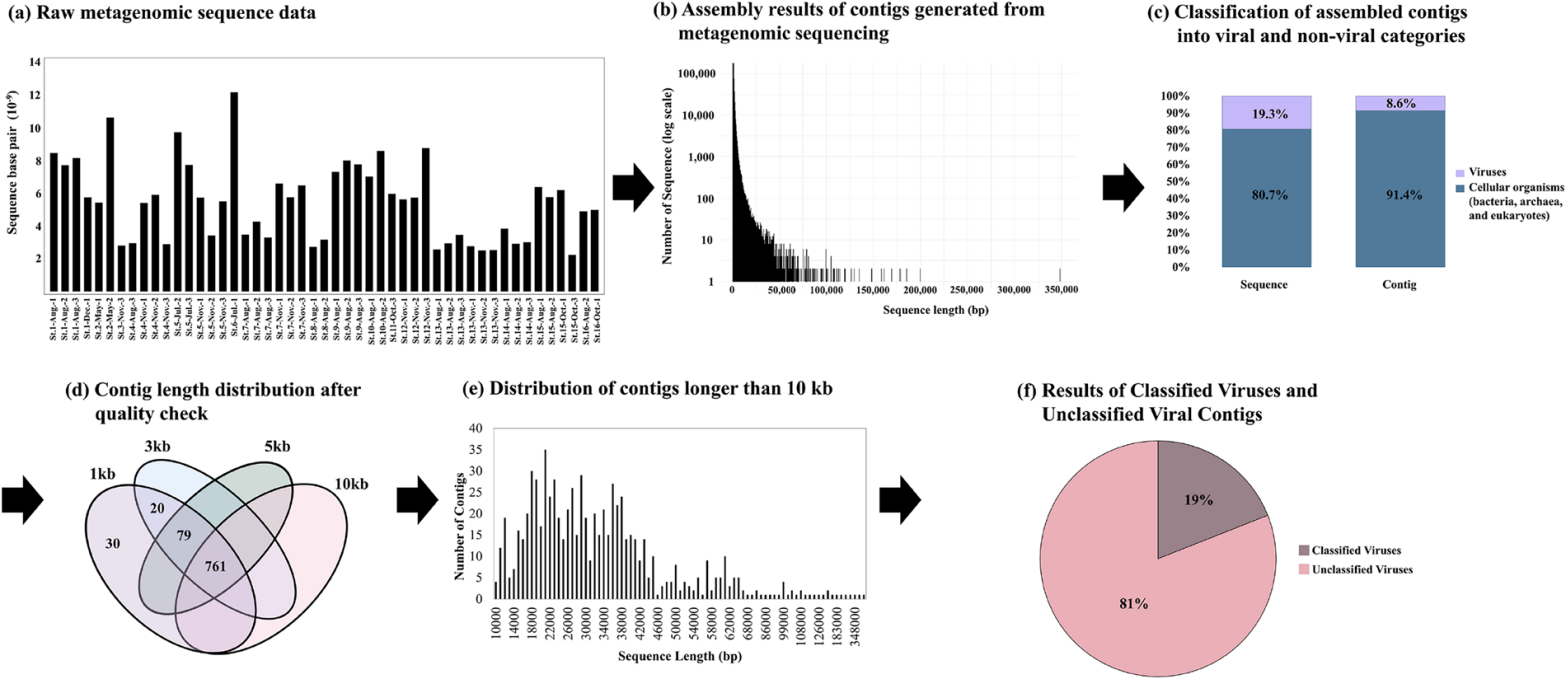
Overview of contig quality check and selection process. (**a**) Raw metagenomic sequence data. (**b**) Assembly results of contigs generated from metagenomic sequencing. (**c**) Classification of assembled contigs into viral and non-viral categories. (**d**) Contig length distribution after quality check. (**e**) Distribution of contigs longer than 10 kb. (**f**) Results of Classified Viruses (e-value: 10^−5^ or Open Ring Frames associated with viral hallmarker gene) and Unclassified Viral Contigs.

**Fig. 3:**
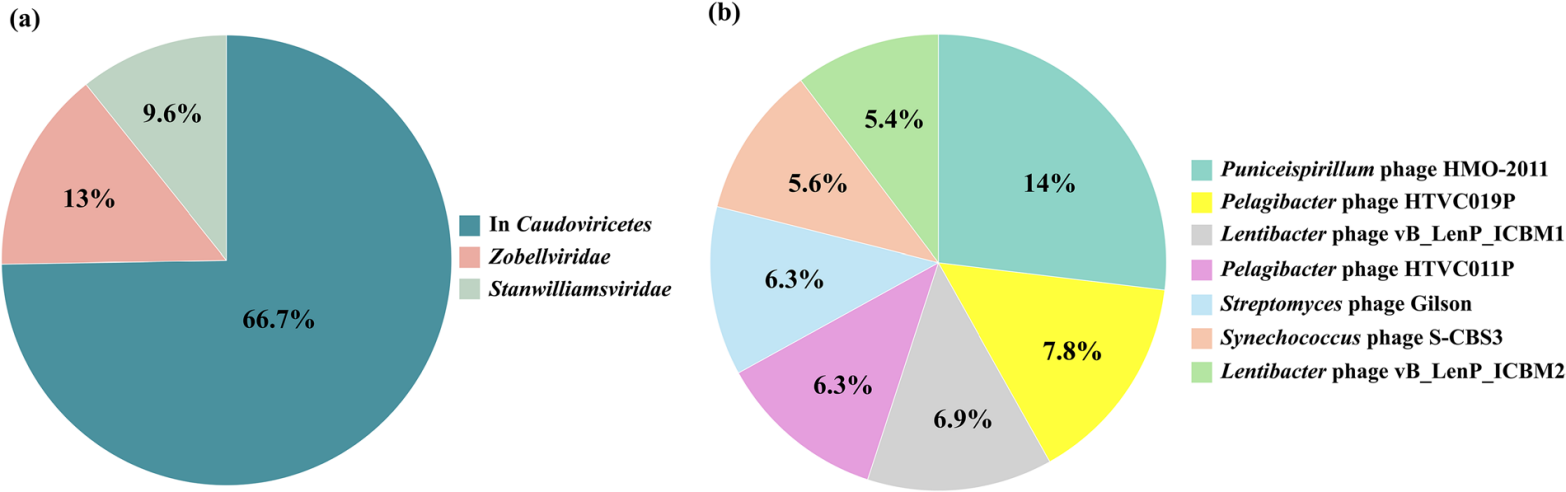
Taxonomic composition of viral contigs based on BLASTn annotation. (**a**) Family-level distribution of taxonomically assigned viral contigs. (**b**) Species-level composition of predominant viral taxa (>5% average abundance).

In this study, surface water samples were collected throughout 2021 from 16 coastal sites around the Republic of Korea (Fig. 1). A total of 265 gigabases of raw sequencing reads were generated from 49 samples, which resulted in 4.06 gigabases of assembled contigs after quality filtering (Fig. 2a,b, Table S1). Quality assessment and classification using CheckV categorized the contigs into viral (19.3%) and non-viral (80.7%) groups (Fig. 2c). Contigs of medium or higher quality were filtered based on length thresholds, resulting in 860 contigs ≥3 kb, 840 contigs ≥5 kb, and 754 contigs ≥10 kb. The average length of contigs ≥10 kb was 36,436 base pairs (Fig. 2d,e). Under these stringent thresholds, 19% of the contigs were successfully taxonomically assigned, while the remaining 81% were classified as unassigned, likely due to the lack of significant homology to known viral sequences (Fig. 2f). At the class level, all bacteriophages were classified as *Caudoviricetes* (Table S2). At the family level, unclassified bacteriophages constituted the largest proportion (66.7%), followed by *Zobellviridae* (13%) and *Stanwilliamsviridae* (9.6%) (Fig. 3a). Seven bacteriophages (*Puniceispirillum* phage HMO-2011, *Pelagibacter* phage HTVC019P, *Lentibacter* phage vB_LenP_ICBM1, *Pelagibacter* phage HTVC011P, *Streptomyces* phage Gilson, *Synechococcus* phage S-CBS3, and *Lentibacter* phage vB_LenP_ICBM2) and *Micromonas pusilla virus* 12 T and SP1 of *Phycodnaviridae* (NCLDVs) were predominant at the species level.

Environmental parameters observed during the sampling period are described on Figshare^24^. In terms of environmental factors, the water temperature range from11.26–34.03 °C (mean ± Standard deviation: 21.8 ± 5.8 °C). The mean salinity was 30.34, excluding Cheonsuman (St. 13, mean salinity 2.68), a freshwater lake connected to the Cheonsuman coastal area, and Eulsukdo (St. 4, mean salinity 13.08), an estuarine area influenced by freshwater input from the Nakdong River. The concentrations of dissolved inorganic nitrogen (DIN) and dissolved inorganic phosphorus (DIP) were 21.48 ± 22.98 μM and 0.92 ± 0.91 μM, respectively. Chlorophyll-*a* concentrations ranged from 0.58 to 37.2 μg L^−1^, with monthly mean exceeding 5 μg L^−1^ in April - May and November - December. Dissolved organic carbon (DOC) concentrations ranged from 1.00 to 5.73 mg L^−1^, with higher values observed during periods of elevated chlorophyll-*a* concentrations.

## Methods

### Sample collection and environmental measurement

To investigate seasonal variation in coastal viral communities and environmental factors, 49 surface seawater samples were collected from 16 sites in Korean coastal waters in 2021 (Fig. 1). At each sampling site, a total of 60 L seawater samples were collected using a Niskin water sampler (General Oceanics Inc., Miami, FL, USA) and transferred to pre-cleaned polyethylene (PE) bottles. Immediately after collection, samples were maintained at 4 °C and transported to the laboratory for further analysis.

The methodology for assessing environmental parameters and biological factors was based on our previous studies^25–27^. Environmental variables, including temperature and salinity, were measured at each sampling site using a multiparameter water quality sonde (EXO2, YSI Inc., Yellow Springs, OH, USA). To ensure data accuracy and cross-validation, three EXO2 multi-parameters were deployed and operated simultaneously. For dissolved inorganic nutrients, dissolved organic carbon (DOC), and chlorophyll-*a* (Chl-*a*) analyses, 2 L of seawater were collected from each site into sterile polyethylene (PE) containers, stored in ice-cooled boxes, and transported to the South Sea Research Institute, KIOST. A 50-mL subsample was filtered through a 47-mm GF/F filter (Whatman, Clifton, NJ, USA) under gravity. The resulting filtrates were transferred into acid-washed PE bottles and either immediately analyzed or stored at –80 °C for no longer than 7 days prior to analysis. Dissolved inorganic nitrogen (NO₂ + NO₃ + NH₄^+^) and dissolved inorganic phosphorus concentrations were determined using a QuAAtro39 continuous flow analyzer (SEAL Analytical, UK). DOC concentrations were quantified via high-temperature catalytic oxidation using a TOC-VCPH analyzer (Shimadzu, Kyoto, Japan). To determine Chl-*a* concentrations, 1 L of seawater was filtered through a GF/F membrane filter and extracted in 90% acetone under dark conditions at 4 °C for 24 hours. Chl-*a* concentrations were then measured using a fluorometer (Trilogy; Turner Designs, Sunnyvale, CA, USA). All measurements were conducted in triplicate to ensure reproducibility.

### Virus flocculation, resuspension, DNA extraction, and sequencing

**Fig. 4:**
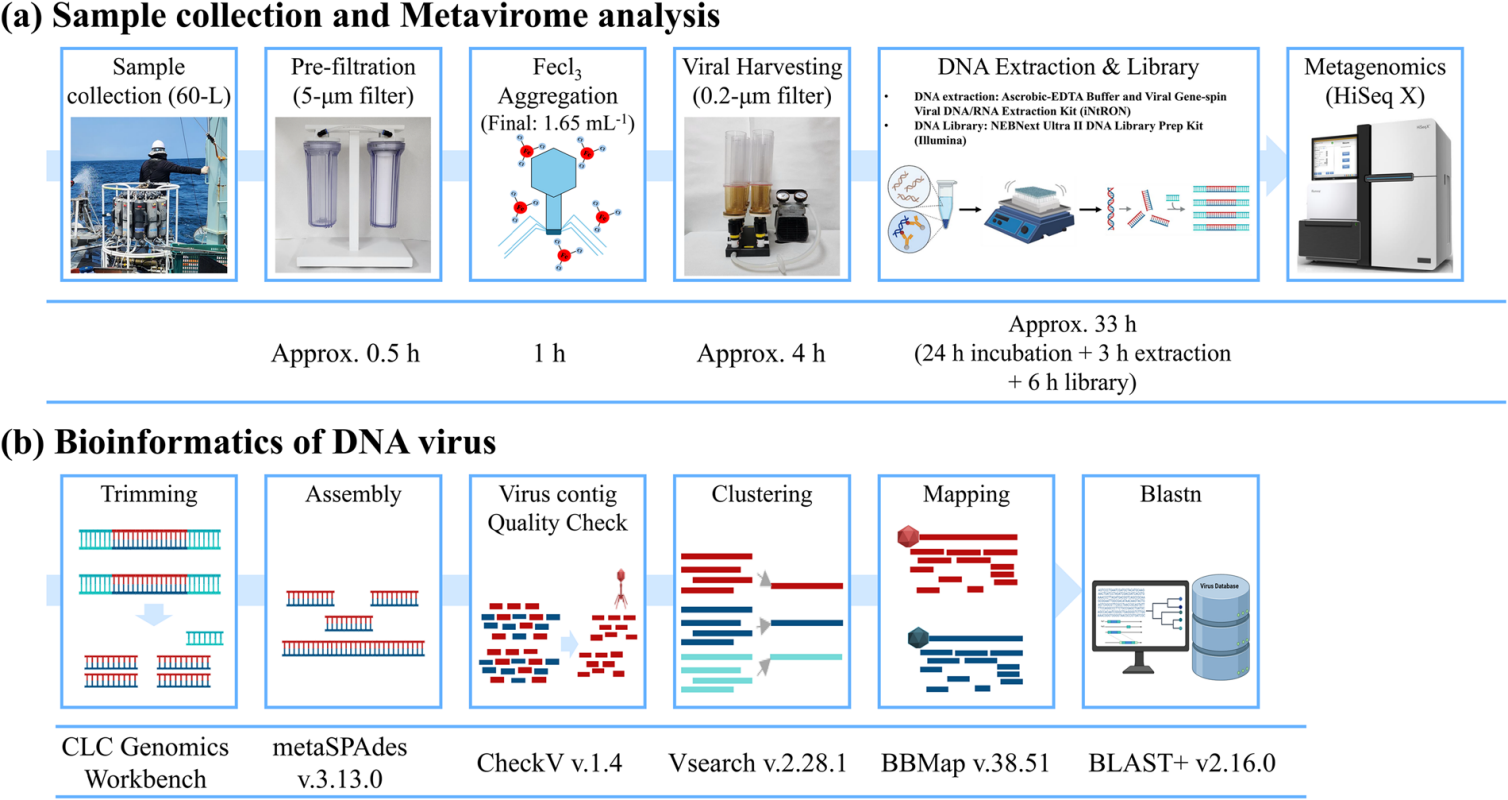
Workflow for DNA Viral metavirome Analysis. (**a**) Sample collection to metavirome analysis. (**b**) Bioinformatics of DNA viral contigs.

Marine viral collection methods include ultracentrifugation^28^, filtration using ultrafine membranes (<0.2 µm)^29^, and aggregation with iron ions (Fig. 4). Among these, Fe-based virus flocculation, filtration, and resuspension (FFR) is highly efficient (>90% recovery), cost-effective, and reliable, making it suitable for studies on marine viral ecology and genomics^30^. In this study, a modified FFR method was applied to analyse marine DNA virus communities^31^. To extract viral genomic DNA (gDNA), 20-L of seawater was filtered through a 5-μm membrane (TMTP04700; Merck Millipore, MA, USA) to remove large organic and inorganic particles. Viruses were concentrated via flocculation using Fe_3_^+^ ions and collected on a 0.2-μm polycarbonate membrane (111106; Whatman, Buckinghamshire, UK), which was stored at 4 °C. Most experiments were performed in triplicate. Following a previously established protocol^27^, we prepared an FeCl₃ solution containing 16.53 mg Fe³^+^ mL^−1^, and added 1 mL of this solution per 10 L of seawater to induce virus particle flocculation. For DNA extraction, the membrane was cut into small sections and placed in a suspension buffer (10 mL of 0.1 M EDTA, 0.2 M MgCl_2_, 0.2 M Ascorbate) in a 50-mL conical tube (Fig. 4). Viruses were released by suspending them in the buffer, and the pH was adjusted to 6 with approximately 5 mL of 10 M NaOH solution. Total gDNA was extracted using the Viral Gene-spin Viral DNA/RNA Extraction Kit (iNtRON Biotechnology, Seoul, South Korea). The extracted gDNA was used to construct a metavirome library with the NEBNext Ultra II DNA Library Prep Kit (Illumina, San Diego, CA, USA), involving random DNA fragmentation, 5′ and 3′ adapter ligation, and amplification via polymerase chain reaction. The prepared library was then sequenced using the Illumina HiSeq X platform in paired-end mode.

### Bioinformatic analyses of DNA viruses

Bioinformatics analysis was conducted using a modified protocol^32,33^ (Fig. 4). Raw sequencing data were processed using CLC Genomics Workbench v20.0.4 (Qiagen, Hilden, Germany), with low-quality reads and sequencing adaptors removed during the preprocessing step. De novo assembly of viral contigs was performed using metaSPAdes v3.13.0^34^ with the following command: ‘metaspades.py -k 21,33,55,77,99,127–pe1-1 R1.fastq–pe1-2 R2.fastq -t 32 -o output_dir’. Assembly quality was assessed using CheckV v1.4^35^ (‘checkv end_to_end input_dir output_dir -t 32’), applying a minimum contig length threshold of 10 kb and included only medium-quality or higher sequences (Table S1, S3). Contig length distributions (≥3 kb, ≥5 kb, and ≥10 kb) are summarized in Table S1. Contigs were dereplicated and clustered at ≥95% average nucleotide identity (ANI) using VSEARCH v2.28.1^36,37^ with ‘vsearch–cluster_size Sample.fa–id 0.95–strand both–sizein–sizeout–fasta_width 0–uc output.uc–centroids output.fa’. Mapping was performed with BBMap v38.51^38^ (‘bbmap.sh in1 = R1.fastq in2 = R2.fastq covstats = output.txt’) at 95% identity, followed by removal of sequencing adapter sequences and PhiX174 control phage contamination. Taxonomic annotation was conducted using BLASTn v2.13.0^39^ against the NCBI Viral RefSeq nucleotide database, with thresholds of e-value ≤ 1e−5^40^, identity ≥ 70%, and bitscore ≥ 50 (Table S4). The BLASTn command used was: ‘blastn -query input.fasta -db RefSeqViral_nucl -evalue 1e-5 -max_target_seqs. 10 -outfmt “6 qseqid sseqid pident length mismatch gapopen qstart qend sstart send evalue bitscore stitle qcovs” -num_threads 16> output.outfmt6’. To complement nucleotide-based classification, BLASTp was also performed against the Viral RefSeq protein database to verify the presence of viral hallmark genes: ‘blastp -query input.fasta -db RefSeqViral_prot -evalue 1e-5 -max_target_seqs. 1 -outfmt “6 qseqid sseqid pident length mismatch gapopen qstart qend sstart send evalue bitscore stitle scomnames” -num_threads 16> output.outfmt6’. Based on the annotation results, contigs were categorized as known viruses (containing annotated viral genes) or unknown viruses (lacking identifiable viral protein matches). In total, 68 viral species were identified under these parameters.

## Data Records

The Illumina sequencing data have been deposited in the NCBI Sequence Read Archive (SRA) under BioProject accession PRJNA1218803^41^. Moreover, the assembled fasta files of viral contigs (≥10 kb) used in this study are deposited in GenBank Nucleotide under accession numbers PV702959 to PV703754. A total of 796 nucleotide sequences (accession numbers PV702959–PV703754) have been uploaded to BioProject PRJNA1218803^41^. In addition, the environmental metadata are publicly available on Figshare (10.6084/m9.figshare.29167460.v1)^42^, and the assembled viral contigs (.fasta) are deposited in a separate Figshare repository (10.6084/m9.figshare.29603600)^24^.

## Technical Validation

### Library quality control

The raw metavirome reads were assessed for quality using the CLC Genomics Workbench v20.0.4 to ensure sequencing data integrity. The calculated Q scores showed that an average of 63.04% to 93.58% of the reads across all sampling sites had a Q30 or higher. These findings suggest that the sequencing data were of high quality, making them appropriate for metagenomic analysis.

### Taxonomic profiling validation

Taxonomic profiling validation confirmed that species-level assignments were consistent and robust. BLASTn classifications were supported by BLASTp results, and contigs containing hallmark or replication-associated genes were reliably classified as known viruses, whereas contigs lacking such genes were categorized as unknown viruses. These results validate the robustness of the taxonomic assignments.

### Replication

To ensure accurate interpretation and reproducibility, three replicate (each 20 L) seawater samples were collected at each sampling site. Independent sequencing libraries were prepared for each replicate and subjected to separate quality control (QC), with only those passing QC included in the analyses. For downstream analyses, the triplicate datasets were processed to calculate the mean and standard deviation, which were then used for interpretation.

## Supplementary information

Supplementary Table S1

Supplementary Table S2

Supplementary Table S3

Supplementary Table S4

## Data Availability

The raw sequencing reads generated in this study are deposited in the NCBI Sequence Read Archive under BioProject accession PRJNA1218803 (SRA Study SRP565150)^41^, comprising 18 BioSamples and 117 SRA runs. The assembled viral contigs (≥10 kb, n = 796) are available in GenBank under accession numbers PV702959–PV703754, all linked to BioProject PRJNA1218803^41^. The environmental metadata and assembled viral contigs are deposited in a Figshare repositories^24,42^.

## Code availability

The newly developed code was not used in this study.
